# Complementary Therapies and Their Association with Problems in Therapeutic Adherence to Conventional Synthetic DMARDs in Rheumatoid Arthritis: A Cross-Sectional Study

**DOI:** 10.3390/healthcare12010049

**Published:** 2023-12-25

**Authors:** Aline Priscilla Santiago-Garcia, Jorge Ivan Gamez-Nava, Felipe Alexis Avalos-Salgado, Sergio Cerpa-Cruz, Edna Lizeth Amaya-Cabrera, Sergio Gutierrez-Ureña, Cesar Arturo Nava-Valdivia, Sergio Gonzalez-Vazquez, Diana Esperanza Arevalo-Simental, Jose Jorge Gomez-Camarena, Juan Manuel Ponce-Guarneros, Norma Alejandra Rodriguez-Jimenez, Ana Miriam Saldaña-Cruz, Ernesto German Cardona-Muñoz, Laura Gonzalez-Lopez

**Affiliations:** 1 Programa de Doctorado en Farmacologia, Centro Universitario de Ciencias de la Salud, Universidad de Guadalajara, Guadalajara 44340, Mexico; aline.santiago@investigacionmedica.com.mx (A.P.S.-G.); ivangamezacademicoudg@gmail.com (J.I.G.-N.); felipe.asalgado@alumnos.udg.mx (F.A.A.-S.); sergtzur2@gmail.com (S.G.-U.); diana.arevalo4984@academicos.udg.mx (D.E.A.-S.); jose.gomez0938@alumnos.udg.mx (J.J.G.-C.); juan.ponce4091@academicos.udg.mx (J.M.P.-G.); norma.rodriguezj@academicos.udg.mx (N.A.R.-J.); ana.saldanac@academicos.udg.mx (A.M.S.-C.); 2 Research Group for Factors Related to Therapeutic Outcomes in Autoimmune Diseases, Centro Universitario de Ciencias de la Salud, Universidad de Guadalajara, Guadalajara 44340, Mexico; cardona@cucs.udg.mx; 3 Programa de Maestria en Salud Publica, Departamento de Salud Pública, Centro Universitario de Ciencias de la Salud, Universidad de Guadalajara, Guadalajara 44340, Mexico; 4 Departamento de Fisiología, Centro Universitario de Ciencias de la Salud, Universidad de Guadalajara, Guadalajara 44340, Mexico; 5 Departamento de Reumatología, Hospital Civil Fray Antonio Alcalde, Guadalajara 45019, Mexico; sacer04@prodigy.net.mx (S.C.-C.); edna.amaya.cabrera@gmail.com (E.L.A.-C.); 6 Departamento de Microbiología y Patología, Centro Universitario de Ciencias de la Salud, Universidad de Guadalajara, Guadalajara 44340, Mexico; cesar.navavaldi@academicos.udg.mx; 7 Hospital General Regional 110 Instituto Mexicano del Seguro Social, Guadalajara 44716, Mexico; sergiogonvaz@yahoo.com.mx

**Keywords:** rheumatoid arthritis, complementary therapies, conventional synthetic disease modifying anti-rheumatic drugs, therapeutic adherences, Morisky–Green scale, cross-sectional study

## Abstract

The use of complementary therapies is highly prevalent among patients with rheumatoid arthritis (RA). Nevertheless, the use of complementary medicine could involve problems in the following of scientifically accepted treatments. To date, there is limited information regarding the association of nonconventional therapies with problems regarding compliance with the treatment. Therefore, the objective of this study was to identify whether the utilization of complementary therapies is associated with a high risk of problems regarding therapeutic adherence to conventional synthetic disease-modifying anti-rheumatic drugs (cs-DMARDs) in RA patients. A survey was performed with RA patients in an outpatient rheumatology clinic in a university hospital; the use of complementary therapies, as well as their type, was identified. To assess problems with therapeutic adherence, we used the four-item Morisky–Green scale. A comprehensive assessment of clinical and therapeutic characteristics was performed. Univariable and multivariable models were performed to identify the risk of problems with therapeutic adherence in users of complementary therapies. In total, 250 RA patients were included; 92% used complementary therapies. Of them, the most frequently used were herbal medicine (65%), homeopathy (64%), and cannabis and its derivatives (51%). In the univariable logistic regression analysis, the factors associated with problems in the therapeutic adherence to cs-DMARDs were age (*p* = 0.019), the presence of other comorbidities (*p* = 0.047), and the use of complementary therapies (*p* = 0.042). After controlling for potential confounders, the use of complementary therapies increased the risk of problems with therapeutic adherence to cs-DMARDs (adjusted OR = 2.84, 95% CI = 1.06–7.63, *p* = 0.037). We concluded that the use of complementary therapies increases the risk of problems with therapeutic adherence. Therefore, for physicians and healthcare professionals, the early identification of the use of nonconventional therapies in their RA patients is required, followed by a directed discussion with their patients about the risks and benefits to which they could be exposed to complementary therapies.

## 1. Introduction

Rheumatoid arthritis (RA) is a chronic inflammatory autoimmune systemic disease that involves synovial joints and leads to severe pain, swelling, and articular damage [1]. The prevalence of RA is 1% worldwide and around 1.6% of the Mexican population [1,2]. RA has been considered the main autoimmune rheumatic disorder observed by rheumatologists in public insurance hospitals in Mexico, being associated with functional impairment, frequent sick leave episodes, and high rates of disability pension [3,4]. Different clinical practice guidelines, such as those of the American College of Rheumatology and the European League for Arthritis and Rheumatism (EULAR), have recommended the early onset of treatment with conventional synthetic disease-modifying antirheumatic agents (cs-DMARDs) once RA is diagnosed, with the objective of achieving an adequate control of the disease [5,6]. However, a significant problem for the adequate therapeutic control of the patients faced by the clinicians is the frequent withdrawal of the medications as well as a limited adherence to the medical prescriptions. The problem of poor adherence to long-term therapies is not privative of RA patients, and it has been reported in a significant number of chronic disorders [7]. However, in RA, the poor therapeutic adherence to ce-DMARDs has been estimated to be between 32% and 40% [8]. A wide number of factors related to poor therapeutic adherence have been investigated in RA, concluding that the problem has a multifactorial etiology [8,9,10]. However, only a few studies have investigated the utilization of nonconventional therapies as factor for the behavior of voluntary suspension of cs-DMARDs by RA patients.

Complementary therapies, also known as nonconventional therapies, are defined as any therapy, product, knowledge, belief, or practice not recommended by standard medical practices in healthcare [11,12]. This list of those therapies includes acupuncture, homeopathy, herbal medicine, insect natural products or venoms (honey bee, ant, wasp or scorpion), mind–body therapies, and biomagnetism, among others [12,13]. 

A high prevalence of using complementary therapies has been observed in studies performed in RA and other rheumatic diseases [14,15]. In our country, complementary therapies are usually considered by the population as “natural products” that are thought to be safe and harmless and can be acquired widely without a formal prescription, propitiating their indiscriminate uses in chronic disorders. Additionally, some complementary medicines have limited supervision by the regulatory agencies favoring the presence of some medicines of poor quality, counterfeit products, or complementary medicine adulterated with allopathic drugs (such as non-steroidal anti-inflammatory drugs [NSAIDS], corticosteroids or other substances non-described in their components); and finally, frequently the prescription of complementary therapies is accomplished by incomplete or unreliable information that could mislead patients to a loss of confidence on the conventional scientific treatment, placing their trust in the alternative therapies. Nevertheless, complementary therapies are not harmless, and studies have identified adverse drug reactions linked to the use of complementary and alternative medicine products [16]. However, as described above, a major problem in the treatment of chronic diseases is the high frequency of withdrawals from the treatments. Therefore, a valid question is whether the use of complementary medicine can influence the limited adherence in RA. Therefore, the objective of the present study was to identify whether the utilization of complementary therapies is associated with a high risk of problems of therapeutic adherence to cs-DMARDs in RA patients.

## 2. Patients and Methods

### 2.1. Study Design and Clinical Setting

This study was designed as a cross-sectional study; we included patients with RA who attended an outpatient rheumatology consultation in a university hospital (Hospital Civil Fray Antonio Alcalde) in Guadalajara, Mexico. The referred center attends patients from several provinces of western Mexico (Jalisco, Michoacan, and Zacatecas), and their population usually corresponds to a low economic stratum according to the average monthly income. The study was performed from 30 January 2023 to 30 July 2023.

### 2.2. Inclusion and Exclusion Criteria

We included patients with RA diagnosed by a rheumatologist and meeting the American Rheumatism Association 1987 revised criteria for RA [17]. These patients were ≥18 years old and had a prescription of cs-DMARDs. Patients were excluded if they had overlapping syndrome, cancer, chronic renal failure, inability to answer questionnaires, or if they were using biologic DMARDs. Patients with any condition or disease leading to disability to answer questionnaires were also excluded. 

### 2.3. Ethics

All the study procedures were approved by the research and ethics of the hospital Civil Fray Antonio Alcalde (code of approval: HCG/FAA/CEI-65/23). This research protocol followed the Ethical Principles for Medical Research Involving Human Subjects described in the Helsinki Declaration issued in Fortaleza, Brazil, in 2013 [18]. All the patients included in the study read and signed a voluntary consent form before participating.

### 2.4. Study Development

Two trained researchers (A.S-G, F.A-S) interviewed RA patients attending the University hospital. These interviews with RA patients were conducted prior to their medical consultation. The survey included an assessment of:

(a) Sociodemographic variables: female gender, age, years of formal education, coupled, non-salaried worker.
(b) Comorbid diseases: depression, hypertension, diabetes mellitus type 2, other comorbid diseases.
(c) Disease characteristics: disease duration, DAS28-ESR (disease activity score of 28 joints—erythrocyte sedimentation rate), HAQ-DI (health assessment questionnaire—disability index).
(d) Pharmacological treatment (cs-DMARDs use, methotrexate, sulfasalazine, antimalarials, other cs-DMARDs, combined cs-DMARDs therapy—use of 2 or more cs-DMARDs simultaneously—and glucocorticoids prescription).

## 3. Assessment of Complementary Therapies Used to Treat RA

We designed a structured questionnaire to identify whether the patients used complementary therapies to treat RA. Regarding the type of complementary therapy, patients were asked if they used any of the following therapies: (A) homeopathy, (B) acupuncture, (C) any herb-based product (phytotherapy), (D) cannabis therapies (included any therapy in which cannabis was ingested, smoked, or topically administered with the aim of improving RA symptoms), (E) counterfeit medicine products (included garlic-based products, dioxide chloride, and other products that had not been approved by regulatory agencies for RA), (F) utilization of biomagnetic therapy chiropractic, (G) massages, (H) spiritual healing (including: mind and body therapies utilized with “curative objectives of arthritis” such as, yoga, meditation, aromatherapy, or Tai Chi), and lastly, (I) utilization of insect or snakes derivatives (included those labeled as “venoms” of scorpions, bees, or parts of snakes, or ants used for treatment of RA symptoms).

## 4. Assessment of Therapeutic Adherence

Therapeutic adherence to cs-DMARDs was assessed by using the four-item Morisky–Green Medication Adherence Scale (MMAS-4 ©) [19]. Several studies performed on RA patients have used the Morisky–Green scale, a questionnaire that has been shown to be a useful, reliable, and valid measure of adherence to treatment in RA patients and other rheumatic disorders [20,21]. This tool asks each patient a series of four Yes or No questions: (1) Do you ever forget to take your medicine? (2) Do you ever have problems remembering to take your medication? (3) When you feel better, do you sometimes stop taking your medicine? (4) Sometimes, if you feel worse when you take your medicine, do you stop taking it? The resulting score ranges from zero to four correct answers. In order to consider patients as fully adhering to their pharmacological treatment, the responses to those questions must follow the next order: No/Yes/No/No; otherwise, the patient is considered to have low/intermediate (incomplete) therapeutic adherence. Therefore, this study considered two groups: (a) RA patients with high adherence to cs-DMARDs) (score = 4 in the MMAS-4 ©, which is considered as adequate adherence) and (b) RA patients with low/intermediate therapeutic adherence to cs-DMARDs) (score ≤ 3 in the MMAS-4 ©). Note: the MMAS-4 scale, content, name, and trademarks are protected by US copyright and trademark laws. Permission for use of the scale and its coding is required. A license agreement is available from MMAR, LLC. (Goodyear, AZ, USA), www.moriskyscale.com (accessed on 15 January 2023) (see information in other acknowledgments).

## 5. Statistical Analysis

The prevalence rates of using complementary therapy and the level of therapeutic adherence (low/intermediate or high adherence) were computed. Independent Student’s *t*-tests were used for comparisons of quantitative variables between groups; chi-square tests (or Fischer exact tests if required) were used for comparisons of proportions between groups. A logistic regression analysis was performed to compute the variables associated with inadequate adherence (low/intermediate adherence) as dependent variables; we included as covariates those variables with a *p*-value < 0.2 in the univariate analysis or those with a potential biological plausibility for explaining the inadequate adherence to cs-DMARDs. Odds ratios (ORs) and their 95% confidence intervals (95% CIs) were calculated for low/intermediate therapeutic adherence to cs-DMARDs; each factor was computed as a measure of risk in these models using the stepwise method. Adjusted ORs (aOR) were obtained in the final multivariable model. The significance level was set at *p* ≤ 0.05. The analyses were performed using the statistical software SPPS Statistics Version 24.

## 6. Results

In total, 268 RA patients were screened. Of these, 18 (6.71%) patients were excluded for the following reasons: unwilling to participate, 11; cancer, 1; non-prescription of cs-DMARDs, 3 patients; and by using concurrently biologic DMARDs, 3 patients. Table 1 describes the sociodemographic and epidemiological characteristics of the patients included in this study. In total, 250 patients with RA were included; of them, 229 (92%) were women. The mean age was 52 ± 13, with a mean disease duration of 11 ± 9 years; two-thirds of the patients (70%) reported not having a paid job at the moment of the study; in addition, almost all (92%, 95% CI 87.91, 95.05%) of the patients reported using complementary therapies. Comorbid diseases were observed in 196 patients (79%); the most common of these were depression (54%), hypertension (30%), and diabetes mellitus type 2 (17%). Regarding disease characteristics, the average value of disease activity assessed by DAS28-ESR was 3.8 ± 1.2, and a HAQ-DI had a median of 0.3 (range 0.0, 2.8). All patients used cs-DMARDs; the most frequently used were Methotrexate (85%), Sulfasalazine (68%), and antimalarials (42%). Lastly, one of two patients was nonadherent to their pharmacological treatment.

Table 2 shows that almost all the patients reported using at least 1 complementary therapy (92%) since their RA diagnosis, with a median number of therapies used of 4 and a maximum of 37. Two-thirds (65.2%) of the patients with complementary therapies used reported using 5 or more. The most frequent ones reported were phytotherapy or herb-based medicines (65%), homeopathy (64%), cannabis and its derivatives (51%), counterfeit medicine (48%), and acupuncture (27%).

Table 3 describes the association of patients scoring low/intermediate (inadequate) adherence to cs-DMARDs with clinical variables. An association between inadequate adherence to cs-DMARDs and the use of complementary therapies was observed (*p* = 0.03). A trend that did not achieve statistical difference was observed between the presence of chronic comorbidities and low/intermediate therapeutic adherence (*p* = 0.08). We did not identify any other association with the rest of the variables investigated.

Additionally, we tested whether patients using acupuncture had differences related to the frequency of inadequate therapeutic adherence compared to users of other types of CAMs. Of 68 patients treated with acupuncture, 27 (39.7%) were nonadherent compared to 161 patients using other types of CAMs; of them, 95 (59%) had inadequate adherence (*p* = 0.007).

Table 4 shows the results of a multivariable logistic regression analysis to identify the risk factors of inadequate adherence (low/intermediate therapeutic adherence to cs-DMARDs) in RA patients. We found as risk factors the following variables: age (OR = 1.02, 95% CI = 1.01–1.05, *p* = 0.019), presence of chronic comorbidities (OR = 1.95, 95% CI = 1.01–3.77, *p* = 0.047), and the use of complementary therapies (OR = 2.85, 95% CI = 1.04–7.84, *p* = 0.042). Using the stepwise method after adjusting by confounders, such as years of formal education, activity of the disease, functional capacity, age, and presence of comorbidities, the only risk factor for an inadequate adherence (low/intermediate therapeutic adherence) was the use of complementary therapies (adjusted OR = 2.84, 95% CI = 1.06–7.63, *p* = 0.037).

## 7. Discussion

This study identified that RA patients who were users of complementary therapies had an increased risk of presenting a low/intermediate therapeutic adherence to cs-DMARDs. Using a multivariable approach, after adjusting for confounders, the use of complementary therapies was found to increase the risk of low/intermediate therapeutic adherence to cs-DMARDs almost 3-fold.

### 7.1. Utilization of Complementary Therapies’ Prevalence and Types

In the present study, we identified a high prevalence of the use of complementary therapies (92%) in our RA patients. Several works have observed wide differences in their studies aiming at the prevalence of using complementary therapies in patients with rheumatic diseases varying from 60% to 99% [22,23,24,25,26,27]. Differences in the prevalence of using complementary or alternative therapies in the different reports depend on multiple factors, including characteristics of the patients such as race, ethnic background, cultural origins, etc. Other factors that might influence the result of prevalence across the studies include differences in their definition of what they considered as complementary therapies as well as the scope of therapies being assessed by each study. In our study, we investigated a wide spectrum of nonconventional therapies. We observed that the most frequent complementary therapies used by our patients were: (a) herb medicine (phytotherapy) (65%), (b) homeopathy (64%), (c) cannabis and its derivatives (51%), (d) counterfeit medicines (48%), and (e) acupuncture (27%). Regarding herb medicine, similar to our results, Rambod M et al. [25], in a study performed on Iranian RA patients, observed that around 51% used herbal medicine. Instead, the use of herb medicine to treat RA in other countries is less frequent. In the United States, it has been reported a lower frequency (19%) of using herbal medicine in patients with RA of race African Americans [14]. Preferences of other types of complementary therapies have been reported in Asian patients with RA; for instance, Han M et al. [26] found in Korean RA patients that acupuncture was the most prevalent complementary therapy used, whereas only 17% used herb medicine. On the other side, in our patients, the second most frequently used CAM was homeopathy. More than a decade ago, it was reported in our city and province (Guadalajara, Jalisco, Mexico) that patients with rheumatic disorders have a high frequency of using homeopathy [27]. However, reports in other provinces of Mexico have reported a lower frequency of homeopathy utilization [23,24]. These variations across the different provinces in Mexico underline the cultural complexity and differences of different provinces in Mexico regarding the preferences for using complementary and alternative medicines. Our results also denote that a high proportion (around half) of patients with RA report the use of cannabis or derives for treating RA mainly chronic pain and other symptoms such as articular stiffness. Previous studies have reported that the use of cannabis as a complementary therapy is frequent in Mexican patients with rheumatic disorders. Caballero et al. reported the use of Cannabis in 30% of their patients. However, 90% of them used it topically, and only 10% used it in a systemic way [24]. A similar prevalence (30%) was reported previously by Ramos-Remus et al. in our province in patients with rheumatic disorders [27]. However, according to our data, the prevalence of using cannabis as complementary therapy in RA seems to be increasing. Regarding acupuncture, only one of every four patients with RA has used acupuncture to treat their symptoms; that prevalence is considerably higher than the previously reported in Mexico for RA patients, with a range from 11% to 14% [23,24,27]. However, in Asian studies, the prevalence of using acupuncture for RA is considerably higher, being reported in around 55% [26].

### 7.2. Prevalence of Low/Intermediate Therapeutic Adherence (Inadequate Adherence)

In the present study, low/intermediate therapeutic adherence to cs-DMARDs was observed in 51% of RA patients. These findings were similar to those previously reported in the literature. Smolen, J. S. et al. examined therapeutic adherence to cs-DMARDs in a large cross-sectional study that included RA patients from Europe, Canada, Latin America, the Asia–Pacific region, and the Middle East, observing that 43% of them were not highly adherent [20]. Wide variations in the optimal full adherence to cs-DMARDs have been reported in different studies. Prudente et al. reported that the prevalence of adherence to medication in their Brazilian RA patients, using the Morisky–Green scale, was only 16.4% of their patients [21]. Instead, Marras, C. et al. found that 29.9% of their Spanish RA patients were nonadherent [28]. Those data highlight the high frequency of inadequate adherence to conventional treatments for RA and the relevance of detecting the problem of non-compliance with the treatment as a potential cause of poor therapeutic response in RA.

### 7.3. Association between Complementary Therapies and Low/Intermediate Therapeutic Adherence

We investigated whether the use of complementary therapies constitutes a risk factor for problems in therapeutic adherence. After controlling for a wide number of confounding factors, this study identified a high risk for reduced compliance to continue taking cs-DMARDs associated with the use of complementary therapies. To our knowledge, only a few works have reported the risk of low adherence to the treatments with the use of nonconventional medicine in RA patients. In the study performed by Westhoff, G. et al. reported that RA patients with preferences for using alternative therapies had an increased risk of cs-DMARDs abstention [29]. Additionally, Lahiri, M. et al. [30] identified that the use of complementary therapies was associated with a delay in cs-DMARDs initiation in RA patients. These data are relevant to identify the potential risk of using complementary therapies without appropriate information on the consequences of abandoning conventional therapy with recognized evidence of effectivity in the control of RA. Finally, our data underline the importance of identifying the problems in therapeutic adherence in RA patients. Morisky–Green scale is a well-known scale used for adherence, showing that it is valid and reliable for detecting problems with therapeutic compliance [19,31,32]. Differences between the use of specific types of CAMs and the frequency of inadequate therapeutic adherence have been found. We identified in this study that acupuncture was associated with a lower prevalence of inadequate therapeutic adherence compared to other CAMs. However, the prevalence observed of inadequate therapeutic adherence was important (39%).

## 8. Strengths and Limitations

The present study presents relevant information on the risk of inadequate therapeutic adherence associated with the use of complementary therapies by using an adjusted multivariate analysis to exclude potential confounders. This study included a wide scope of complementary therapies, identifying that the major complementary therapies used by RA patients in our setting are herbal medicine, homeopathy, and cannabis and its derivatives. Additionally, it underlines that the use of cannabis to treat difficult symptoms of RA is frequent in our patients.

However, our study has several limitations: firstly, it is a cross-sectional study constituting a snapshot of the prevalence of the use of complementary therapies. That prevalence changes over time. Therefore, a prospective cohort study will be required to identify changes in the preferences of the type of complementary therapies during the disease evolution and consider periods with variation in the response to conventional accepted therapies. Secondly, there is a certain reluctance on the part of patients to recognize the use of these therapies. Therefore, a validated questionnaire is necessary to identify them. Third, it is necessary to include in the programs of health education directed to RA patients to include teaching the pros and cons of using complementary therapies and inform their physicians regarding the use of these therapies as well as the potential impact on the health of dropping their conventional, accepted therapies for controlling the disease. Finally, a future issue is working to standardize the classification of these types of therapies in order to compare the results in different studies.

## 9. Conclusions

According to our data, around one in two RA patients has problems in their therapeutic adherence to cs-DMARDs, and one of the main risk factors associated with problems in their compliance to conventional treatments to control their disease is the use of complementary therapies. Physicians and other healthcare professionals must identify the use of nonconventional therapies early in their patients and discuss with their patients the risks and benefits to which they could be exposed and the importance of adhering to their pharmacological treatment, emphasizing that high therapeutic adherence is required to achieve pharmacological treatment goals in RA, including to maintain a low activity and/or remission of the disease.

## Figures and Tables

**Table 1:** Characteristics of patients with rheumatoid arthritis.

	n = 250 (%)
Female gender, n (%)	229 (92)
Age, mean ± SD	52 ± 13
Years of formal education, mean ± SD	8 ± 4
Coupled, n (%)	153 (60)
Nonworker, n (%)	175 (70)
Comorbid diseases, n (%)	196 (79)
Depression, n (%)	135 (54)
Hypertension, n (%)	76 (30)
Diabetes mellitus type 2, n (%)	42 (17)
Other comorbid diseases, n (%)	46 (18)
Disease duration (years), mean ± SD	11 ± 9
DAS28-ESR * score, mean ± SD	3.8 ± 1.2
HAQ-DI ** median (range)	0.3 (0.0, 2.8)
cs-DMARDs use, n (%)	250 (100)
- Methotrexate, n (%)	211 (85)
- Sulfasalazine, n (%)	170 (68)
- Antimalarials, n (%)	104 (42)
- Other cs-DMARDs, n (%)	37 (15)
Combined cs-DMARDs therapy, n (%) *	193 (77.2)
Glucocorticoids prescription, n (%)	58 (22)
Low/intermediate therapeutic adherence (MMAS-4), n (%)	128 (51)
Use of complementary therapy, n (%)	229 (92)

Qualitative variables are expressed as frequency and percentage, quantitative variables as means and standard deviations (SD) or as median with minimum and maximum values. * DAS28-ESR—Disease activity score of 28 joints—erythrocyte sedimentation rate. ** HAQ-DI—health assessment questionnaire—disability index. cs-DMARDs—conventional synthetic disease-modifying anti-rheumatic drugs. MMAS-4—four-item Morisky–Green Medication Adherence Scale.

**Table 2:** Usage of complementary therapies.

	n = 250 (100%)
Use of complementary therapy, n (%)	229 (92)
Herbal medicine, n (%)	163 (65)
Homeopathy, n (%)	159 (64)
Cannabis and its derivatives, n (%)	127 (51)
Counterfeit medicine, n (%)	120 (48)
Acupuncture, n (%)	68 (27)
Chiropractic and massages, n (%)	67 (26)
Spiritual healing, n (%)	57 (23)
Mind–body therapies, n (%)	47 (19)
Biomagnetic therapy, n (%)	43 (17)
Insect products or venoms, n (%)	29 (12)
Number of complementary therapies used, median (min., max.)	4 (0, 37)
- 1–2, n (%)	60 (24)
- 3–4, n (%)	54 (22)
- ≥5, n (%)	163 (65.2)

Qualitative variables are expressed as frequency and percentage, and quantitative variables as medians (ranges).

**Table 3:** Comparison of variables between patients with inadequate adherence versus adherence to cs-DMARDs.

	Low/Intermediate Therapeutic Adherence n = 128	High Therapeutic Adherence n = 122	*p*
Females, n (%)	117 (91)	112 (92)	0.9
Age (years), mean ± SD	51.05 ± 13	53.57 ± 12	0.1
Married, n (%)	76 (59)	75 (62)	0.7
Formal education (yrs.), mean ± SD	7.57 ± 4	8.17 ± 4	0.2
Active worker, n (%)	43 (34)	30 (25)	0.1
DAS-28 ERS score, mean ± SD	3.92 ± 1.17	3.86 ± 1.18	0.6
Disease duration, mean ± SD	10.26 ± 10	10.92 ± 9	0.5
HAQ-DI score test, mean ± SD	0.58 ± 0.71	0.50 ± 0.63	0.3
Chronic comorbidities, n (%)	106 (83)	90 (74)	0.08
Complementary therapies, n (%)	122 (95)	107 (88)	0.030
Num. of CAMs *, median (ranges)	4 (0, 37)	4 (0, 36)	0.3

* CAMs—Complementary or Alternative medicines. Num. of CAMs expresses the number of complementary/alternative medicines used by patients; the ranges express the minimum and maximum Num. of CAMs used by each group of patients according to their therapeutic adherence. Comparisons between proportions were made using chi-square tests; comparisons between means were made with Student’s *t*-tests. Comparisons between medians in the numbers of CAMs used were made with the Mann–Whitney *U* test. DAS28-ESR—disease activity score of 28 joints–erythrocyte sedimentation rate. SD—Standard Deviation. HAQ-DI—health assessment questionnaire—disability index.

**Table 4:** Risk factors for inadequate adherence to treatment (low/intermediate therapeutic adherence) in patients with rheumatoid arthritis.

	Low/Intermediate Therapeutic Adherence to cs-DMARDs					
	Unadjusted			Adjusted		
	Enter Method			Stepwise Method		
	OR	95% CI	*p*-Value	aOR	95% CI	*p*-Value
Age	1.02	1.01–1.05	0.019	--	--	--
Comorbidities	1.95	1.01–3.77	0.047	--	--	--
Formal education	1.06	0.98–1.13	0.1	--	--	--
DAS28-ESR	1.11	0.86–1.45	0.4	--	--	--
HAQ-DI	0.78	0.49–1.23	0.4	--	--	--
Use of CAMs	2.85	1.04–7.84	0.042	2.84	1.06–7.63	0.037

Abbreviations: DAS28-ESR—disease activity score of 28 joints–erythrocyte sedimentation rate. HAQ-DI—health assessment questionnaire—disability index. CAMs—complementary or alternative medicines. aOR—adjusted odds ratio. Multivariate analysis—logistic regression analysis. Dependent variable—inadequate adherence: low/intermediate therapeutic adherence to cs-DMARDs. OR—odds ratio. 95 CI—95% confidence interval. Crude ORs were obtained using the enter method. aOR was obtained using the stepwise method. Variables excluded from the final model were age, comorbidities, formal education, DAS28-ESR, and HAQ-DI. cs-DMARDs—conventional synthetic disease-modifying anti-rheumatic drugs.

## Data Availability

The dataset supporting the conclusions presented in this article is available on request from the corresponding author on reasonable request.
